# Knowledge-based variable selection for learning rules from proteomic data

**DOI:** 10.1186/1471-2105-10-S9-S16

**Published:** 2009-09-17

**Authors:** Jonathan L Lustgarten, Shyam Visweswaran, Robert P Bowser, William R Hogan, Vanathi Gopalakrishnan

**Affiliations:** 1 Department of Biomedical Informatics, University of Pittsburgh, 200 Meyran Ave, Parkvale M-183, Pittsburgh, PA, USA; 2 Department of Pathology, University of Pittsburgh, S-417 BST, 200 Lothrop Street, Pittsburgh, PA 15261, USA; 3 University of Pittsburgh Medical Center, Pittsburgh, PA, USA

## Abstract

**Background:**

The incorporation of biological knowledge can enhance the analysis of biomedical data. We present a novel method that uses a proteomic knowledge base to enhance the performance of a rule-learning algorithm in identifying putative biomarkers of disease from high-dimensional proteomic mass spectral data. In particular, we use the Empirical Proteomics Ontology Knowledge Base (EPO-KB) that contains previously identified and validated proteomic biomarkers to select *m/z*s in a proteomic dataset prior to analysis to increase performance.

**Results:**

We show that using EPO-KB as a pre-processing method, specifically selecting all biomarkers found only in the biofluid of the proteomic dataset, reduces the dimensionality by 95% and provides a statistically significantly greater increase in performance over no variable selection and random variable selection.

**Conclusion:**

Knowledge-based variable selection even with a sparsely-populated resource such as the EPO-KB increases overall performance of rule-learning for disease classification from high-dimensional proteomic mass spectra.

## Background

While biological knowledge is typically used to validate the results obtained from the analysis of high-dimensional biomedical data, increasingly, it is being incorporated into the statistical analysis and modeling of such data. For example, the use of knowledge bases to help process and analyze biomedical data for markers of disease has been shown to produce better results than analyzing such data in isolation [1,2]. Biomedical knowledge bases have been growing in number and coverage; examples of such knowledge bases include Gene Ontology (GO), KEGG, UniProt, and EPO-KB [3-6]. These knowledge bases attempt to organize current knowledge in a machine-parsable and human-understandable form and provide biological knowledge that can be used when inducing models from biomedical data.

We focus here on the analyses of proteomic data obtained from mass spectrometry studies to uncover putative biomarkers of disease. Typically, data mining algorithms are used to analyze mass spectral data to identify mass-to-charge ratios (*m/z*s) that are associated with disease [7,8]. Such analyses involve sorting through thousands of *m/z*s that may or may not have biological significance [9]. There has been prior work in the use biological knowledge to assist in proteomic biomarker identification; typically such knowledge has been used for post-processing *m*/*z*s that have been identified by data mining algorithms. Barbarini, et al. used the data in the Human Plasma Proteome Project (Hupo-PPP) to assign putative identification to *m/z*s by translating the *m/z *to a molecular weight in Daltons [10]. Their main goal was in-silico identification for biomarker discovery using a feature selection algorithm. They suggest that performing a biologically-driven feature selection could be beneficial.

In this paper, we present a novel method that uses a proteomic knowledge base to enhance the performance of a rule-learning algorithm in identifying putative biomarkers in high-dimensional proteomic data. In particular, we use the Empirical Proteomics Ontology Knowledge Base (EPO-KB) that contains previously identified and validated biomarkers to select *m/z*s in a proteomic dataset and show that this knowledge-based selection of *m/z*s improves the performance of the rule-learning algorithm.

## Methods

**Figure 1:**
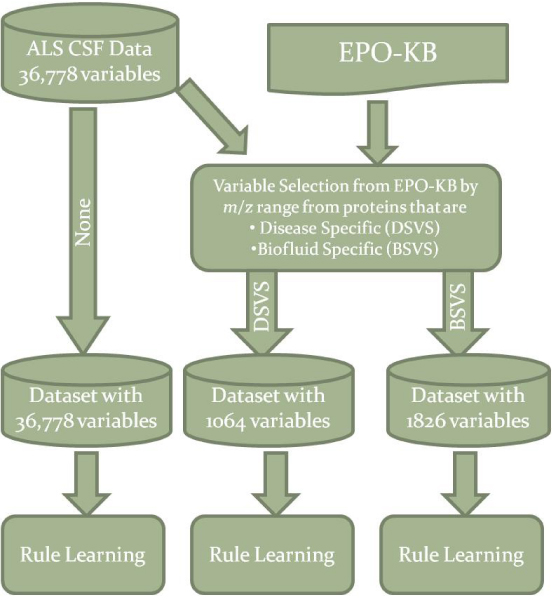
**The methodology for the knowledge-based variable selection showing the three dataset generated**. The three datasets selected by None, DS variable selection, BS variable selection were all subjected to the same rule learning process.

We describe two strategies for knowledge-based biomarker selection in an Amyotrophic Lateral Sclerosis proteomic dataset and evaluate the performance of these strategies on a rule learning algorithm relative to the baseline with to no variable selection. Figure 1 shows a flowchart of the experimental protocol. In the following sections, we first briefly describe the proteomic dataset and the EPO-KB and then describe the variable selection methods, the rule learning algorithm and the evaluation measures.

### Proteomic dataset

We used a proteomic dataset from a study of a rapidly neurodegenerative disease called Amyotrophic Lateral Sclerosis (ALS) where the analyzed samples were obtained from the cerebrospinal fluid (CSF) as described in Ranganathan, et al. [7] The mass spectra from the samples were acquired on a Ciphergen PBSIIc Biomarker Discovery System that performs Surface Enhanced Laser/Desorption Ionization – Time of Flight (SELDI-TOF) mass spectrometry analysis. The dataset has 36,778 *m/z*s at which relative intensities or peaks were measured in a total of 52 samples of which 23 were cases that were obtained from patients diagnosed with ALS and the remaining 29 were controls that were obtained from individuals without ALS.

### The Empirical Proteomics Ontology Knowledge Base (EPO-KB)

A biomarker can be defined as an *m/z *with relative intensity within a specified interval that is associated with a normal biologic process, a pathogenic process, or a pharmacologic response [11]. We have developed a knowledge base called the Empirical Proteomics Ontology Knowledge Base (EPO-KB) that contains biomarkers linked to proteins, peptides and their known modifications as well as associated diseases that we have curated from the literature [6]. In EPO-KB, each protein or peptide is associated with a range of *m/z *values. This is due to the fact that a protein can have closely related *m/z *values in different spectra based on the specific post-translational modifications, the specific biofluid (such as CSF, plasma, nipple aspirate fluid, or tissue lysate) from which it is obtained and the specific method for acquiring the mass spectra (e.g., SELDI-TOF, MALDI-TOF). An example of a biomarker in the EPO-KB is Transthyretin. Transthyretin is associated with the ranges 13727–13766 *m/z *(the singly charged state), 6860–6685 *m/z *(the doubly charged state), and 13873–13923 *m/z *(a post-translational modification). Given an *m/z *value from an experimental spectrum, EPO-KB associates with it proteins and peptides whose ranges of *m/z *values found in the literature include this value. For the experiments described in this paper, we removed those biomarker entries from EPO-KB that were obtained from Ranganathan, et al. [7] so that our analyses simulates using a knowledge base that does not contain knowledge extracted from the dataset being analyzed.

### Variable selection using EPO-KB

We generated three datasets each with different sets of variables. The first dataset included all the variables from the original ALS dataset which we call 'None' for no knowledge-based variable selection. Variables for the second and third datasets were selected in conjunction with EPO-KB. The second dataset included only those variables that were associated with CSF proteins and peptides linked with neurological diseases in EPO-KB (20 proteins and isoforms). The neurological diseases represented in EPO-KB currently include ALS, Alzheimer's disease, and Parkinson's disease and consist of 20 proteins and isoforms that were extracted from four research articles. Thus, the second dataset contained variables that were selected based on their previously being identified in neurological diseases. The third dataset included only those variables that were associated with biomarkers found in CSF in EPO-KB. The biofluids represented in EPO-KB currently include plasma, serum, CSF, urine, and amniotic fluid; the CSF biomarkers include 50 proteins and isoforms that were extracted from seven research articles. Thus, the third dataset contained variables that were selected based on their previously being identified in the same biofluid as found in the ALS dataset. We call the three variable selection methods as no variable selection (None), disease-specific variable selection (DSVS), and biofluid-specific variable selection (BSVS).

### Rule learning

Rule-learning is a useful technique for knowledge discovery from data that is discrete [12-14]. This technique generates a set of propositional IF-THEN rules where a rule consists of an antecedent on its left hand side and a consequent on its right hand side. An example of a rule is:

IF (6889*m/z *= [0.25–0.40]) AND (12282*m/z *= [0.50–0.65]) THEN (ALS = True),

where the values in square brackets represent an interval of relative intensity for the particular *m/z *value. In the above rule, the antecedent consists of two conjuncts, each expressed as a variable-value pair. The first variable-value pair states that the variable 6889 m/z should have a relative intensity value that lies in the range 0.25 to 0.40. The consequent represents the value of the target variable. For a rule derived from proteomic data, the variables in the antecedent represent a potential panel of biomarkers that are discriminative for the target disease [7,8].

We used an algorithm called Rule Learner (RL) for learning sets of IF-THEN rules from the three datasets described in the previous section. The rules learned by RL do not partition the input space as, for example, is done by the classification and regression tree (CART) algorithms; instead they cover overlapping regions in the input space. RL has been used in multiple domains including in novel biomarker discovery, and in parameter learning for protein crystallization [15] and is considered to be an algorithm with low bias and variance [16]. We chose RL for our experiments since it was originally used by Ranganathan et al[7] to analyze the ALS dataset. In our experiments, we constrained RL to learn rules with a maximum of 7 variables in the antecedents to reduce search time and space.

Since RL cannot utilize continuous variables, we discretized the variables using the MDLPC discretization method developed by Fayyad and Irani [17]. This method is widely used for discretization and has been shown to perform well on biomedical datasets [18]. In addition to transforming a continuous variable into a discrete one, the process of discretization also performs variable (feature) selection, since variables that are discretized to a single interval are not predictive of the target variable and are ignored by the rule learning algorithm.

### Measures of performance and statistical analysis

We used balanced accuracy and Relative Classifier Information [19] to measure the classification performance of RL. Balanced accuracy (BACC) is superior to accuracy since it compensates for skewed distribution of classes in a dataset. Balanced accuracy is defined as follows:



where |C| is the cardinality of the target variable and Sensitivity(*c*) (Specificity(*c*)) refers to the sensitivity (specificity) of the target value *c *versus all other values of the target. TP_(c|c) _(True Positives) is the number of samples predicted to have the value *c *for the target variable given that the observed value is *c*, FN_(¬c|c) _(False Negatives) is the number of samples predicted to have a value other than *c *for the target variable given that the observed value is *c*, TN_(¬c|¬c) _(True Negatives) is the number of samples predicted to have a value other than *c *for the target variable given that the observed value is not *c*, and FP_(c|¬c) _(False Postives) is the number of samples predicted to have the value *c *for the target variable given that the observed value is not *c*.

Relative Classifier Information (RCI) is an entropy-based performance measure that quantifies how much the uncertainty of a decision problem is reduced by a classifier relative to classifying using only the prior probability distribution of the values of the target variable uninformed by any predictor variable [19]. The minimum value for RCI is 0% that is achieved when the prediction is always the majority value of the target variable, and the maximum value is 100% that is achieved when the value of the target variable is always correctly predicted. RCI is sensitive to the distribution of the target-values in the dataset and thus compensates for the observation that it is easier to obtain high accuracies on highly skewed datasets.

For comparing the performance of RL on the three variable selection methods, we used the Wilcoxon paired samples signed rank test and the paired samples t-test. The Wilcoxon paired samples signed rank test is a non-parametric procedure that tests whether there is sufficient evidence that the median of two probability distributions differ. The paired samples t-test is a parametric procedure used to determine whether there is a significant difference between the average values for two different sets of samples. The test assumes that the paired differences are independent and identically normally distributed.

### Experiments

To evaluate the performance of RL on a dataset, we performed 10 runs of 10-fold cross-validation to generate a total of 100 folds. We computed the BACC for each fold and averaged over the 100 folds; we report the average BACC in the results. We computed the average RCI in a similar fashion. For the datasets derived using DSVS and BSVS, the BACC and RCI were validated using a randomization test. The randomization test consists of selecting randomly the same number of variables from the original datasets as selected by DSVS (or BSVS), applying RL to the dataset generated using the randomly selected variables, and evaluating its performance in terms of BACC and RCI using 10 runs of 10-fold cross-validation. To perform the randomization test, we generated 1000 datasets by applying random variable selection using the cardinality for each of the DSVS and BSVS methods. In all experiments including the randomization experiments, we utilized the same training and test folds for every dataset. Thus, each of the 100 folds consisted of the same samples (though different sets of variables), irrespective of the variable selection method that was employed.

We also tabulated and compared the *m/z*s that were included in the antecedents of the rules learned by RL on each of the three datasets. For this analysis, we examined the rules generated by applying RL to the entire dataset containing all the samples.

## Results

For both DSVS and BSVS, we have to correlate the protein to a *m/z *range. Utilizing the EPO-KB, we were able to retrieve empirically generated *m/z *ranges (e.g., 13593–13680), and we also included possible double charges when selecting the variables. The original ALS dataset contained 36,778 variables. With the DSVS, we generated 23 ranges (46 with double charges) translating to 1064 *m/z*s selected (3% of the original dataset). For BSVS, since there was an increase in the number of proteins (32 proteins), we generated 64 *m/z *ranges including which lead to a total of 1852 *m/z*s selected (5% of the original dataset). Of note, all of the variables selected by DSVS were also selected by BSVS.

The average BACCs for the different variable selection methods are given in Table 1. The lowest average BACC was on the dataset with no filtering (None). DSVS resulted in an increase in average BACC with associated decrease in sensitivity and increase in specificity over None. BSVS resulted in the highest average BACC with associated increase in sensitivity and no decrease in specificity over None. The increase in average BACC achieved by both methods of knowledge-based filtering of variables over no filtering was statistically significant at the 0.05 level (Table 2).

**Table 1:** Average balanced accuracy (BACC) of the three variable selection methods

Variable selection method	BACC (± std. dev) (Sensitivity, Specificity)	P-Value (randomization test)
None	66.40% (± 19.66) (53.3%, 79.5%)	-
DSVS	71.84% (± 17.49) (50.58%, 93.1%)	0.068
BSVS	78.24% (± 18.48) (66.78%, 89.7%)	**0.003**

None indicates no variable selection, DSVS is disease-specific variable selection and BSVS is biofluid-specific variable selection.

**Table 2:** Statistical comparison of the three variable selection methods using the Wilcoxon Signed Rank test and the t-test on BACC and RCI

Methods	BACC		RCI	
	
	Wilcox	t-test	Wilcox	t-text
None vs. DSVS	**0.011 +**	**0.008 **+	0.074 +	0.065 +
None vs. BSVS	**<0.001 **+	**<0.001 **+	**0.005 **+	**<0.001 +**
DSVS vs. BSVS	**< 0.001 +**	**< 0.001 +**	**0.007 +**	**0.013 **+

p-values below the 0.05 significance level are in bold font. A + favors the second method in a pair.

The average RCI for the various variable selection methods are given in Table 3. The lowest average RCI was obtained by the None method. Though both DSVS and BSVS resulted in higher average RCI than None; the increase associated with DSVS was not statistically significant at the 0.05 level. The highest average RCI was obtained by the BSVS method, which was statistically significant at the 0.05 level (Table 2).

**Table 3:** Average RCI performance of the three variable selection methods

Variable selection method	RCI (std. dev.)	P-Value (randomization test)
None	12.72 (± 2.0)	-
DSVS	15.88 (± 4.3)	0.077
BSVS	20.15 (± 4.3)	**0.001**

On the randomization test, performance of DSVS as measured by BACC and RCI was statisitically significant at the 0.10 level and the performance of BSVS on both BACC and RCI was statisitically significant at the 0.10 level (Tables 1 and 3).

**Figure 2:**
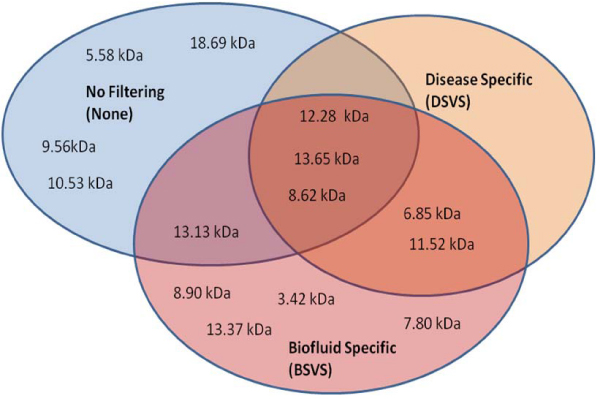
**Venn diagram showing the overlap of the variables selected by RL for the three different variable selection methods**.

The number of variables and the *m/z*s present in the antecedents of the rules learned by RL on the entire dataset for the three variable selection methods are shown in Figure 2. Many more variables were obtained by BSVS as compared to DSVS, and all the *m/z*s obtained by BSVS were also obtained by DSVS. Also, the rules produced by the None method had shorter antecedents (i.e., fewer variables) than either BSVS or DSVS (data not shown).

Table 4 compares the *m/z*s obtained by the three variables selection methods. Fifty percent of the variables obtained by None were different when compared to those obtained by BSVS, and DSVS selected no new variables when compared to those obtained by BSVS.

**Table 4:** *m*/*z*s that were present in the rules for None and DSVS respectively that were not present in the rules for BSVS

Variable selection method	*M/z*s in rules (kDa)	Percent Different
None	5.58, 9.56, 10.53, 18.69	50%
DSVS	None	0%

## Discussion

Proteomic datasets derived from mass spectrometry experiments are typically high-dimensional, noisy, and have small sample sizes. The incorporation of prior biological knowledge can help in statistical analysis and modelingof such datasets where the goal is to identify putative panels of biomarkers associated with disease. We used a database of previously validated biomarkers to perform knowledge-based variable selection to reduce the dimensionality of a proteomic dataset associated with ALS. Our results show that such knowledge-based variable selection can improve the performance of a rule learning algorithm for identifying biomarker panels. We used the *m/z *ranges derived from prior published knowledge to filter the large number of *m/z*s generated in a proteomic dataset using two strategies. First, we used a disease-specific strategy, in which we filtered the *m/z*s based on validated biomarkers associated with neurological diseases; this resulted in increased BACC that was statistically significant at the 5% significance level. Second, we used a biofluid-specific strategy, in which we filtered the *m/z*s by proteins that were present in the CSF; this resulted in increased BACC and RCI that was statistically significant at the 5% significance level. Our approach is similar to the that used in [10] except that instead of using a theoretical database for post-processing, we used an empirical database for preprocessing. We did not use a database like UniProt or Hupo-PPP that contains *m*/*z*s that are theoretically predicted for proteins and peptides for several reasons. First, the theoretical *m/z *is often not predictive of the experimentally derived *m/z*, particularly for those proteins that fragment, cleave, or undergo post-translational modifications. Second, the same protein obtained from different biofluids differs slightly leading to different *m/z*s. Using a database like EPO-KB that contains empirically derived *m*/*z*s allows us to use *m/z*s that have been recorded in previous experiments, which are more reliable than theoretically derived *m/z*s.

One limitation of our study is due to the limited data present in the EPO-KB. Thus, the number of variables selected by DSVS was more than 50% fewer than the number of variables selected by BSVS. In addition, the variables selected by DSVS was a subset of the variables selected by BSVS. The paucity of variables available for DSVS likely resulted in its performance being poorer compared to that of BSVS as well as on the randomization test. In future experiments, it would be interesting to examine if the "intersection" of the knowledge-based biomarker sets provide higher accuracy than the individual biomarker sets. In our current study, since the BSVS set is completely contained in the DSVS set, there is no advantage in using the "intersection" of the two variables sets. Another limitation of our study is that we did not examine the performance of other methods for learning predictive models in addition to the RL algorithm.

Regardless of how one selects *m/z*s for a putative biomarker panel, the *m/z*s have to be identified definitively with techniques such as peptide mass-fingerprinting or tandem mass spectral analyses. A resource like EPO-KB that contains experimentally verified *m/z *to protein associations, is valuable for assigning a small list of putative proteins to *m/z*s of interest in an experiment. While EPO-KB was originally developed for this purpose, this paper demonstrates an additional application of the knowledge in EPO-KB for biomarker analysis of mass spectral data.

## Conclusion

In conclusion, we have presented an example of how even a sparsely-populated knowledge base such as the EPO-KB can be used to perform variable selection to increase classification performance on a high-dimensional proteomic dataset. As more information is added to the EPO-KB, it is expected that such knowledge-based variable selection methods would lead to more useful models. In the future, we will evaluate the combination of knowledge-based biomarkers with dataset specific markers for disease classification.

## Competing interests

The authors declare that they have no competing interests.

## Authors' contributions

JLL, SV, WRH, and VG contributed to designing the experimental design. JLL was responsible for performing the experiments and for writing the first draft of the manuscript. JLL, RPB, WRH contributed to the curation and maintenance of the EPO-KB. RPB generated and characterized the proteomic dataset. VG was responsible for the development of the Rule Learning program as well as oversight over the experiments.

## References

[B1] Tari L, Baral C, Kim S (2008). Fuzzy c-means clustering with prior biological knowledge. Journal of Biomedical Informatics.

[B2] Pan W (2006). Incorporating gene functions as priors in model-based clustering of microarray gene expression data. Bioinformatics.

[B3] Kanehisa M, Goto S, Hattori M, Aoki-Kinoshita KF, Itoh M, Kawashima S, Katayama T, Araki M, Hirakawa M (2006). From genomics to chemical genomics: new developments in KEGG. Nucleic Acids Research.

[B4] UniProt Consortium (2007). The Universal Protein Resource (UniProt). Nucleic Acids Res.

[B5] The Gene Ontology Consortium (2000). Gene Ontology: Tool for the unification of biology. Nature Genetics.

[B6] Lustgarten JL, Kimmel C, Ryberg H, Hogan W (2008). EPO-KB: A searchable knowledge base of biomarker to protein links. Bioinformatics.

[B7] Ranganathan S, Williams E, Ganchev P, Gopalakrishnan V, Lacomis D, Urbinelli L, Newhall K, Cudkowicz ME, Brown RH, Bowser R (2005). Proteomic profiling of cerebrospinal fluid identifies biomarkers for amyotrophic lateral sclerosis. Journal of neurochemistry.

[B8] Gopalakrishnan V, Ganchev P, Ranganathan S, Bowser R (2006). Rule learning for disease-specific biomarker discovery from clinical proteomic mass spectra. Springer Lecture Notes in Computer Science.

[B9] Gopalakrishnan V, Williams E, Ranganathan S, Bowser R, Cudkowic ME, Novelli M, Lattazi W, Gambotto A, Day BW (2004). Proteomic data mining challenges in identification of disease-specific biomarkers from variable resolution mass spectra. Proceedings of SIAM Bioinformatics Workshop.

[B10] Barbarini N, Magni P, Bellazzi R (2006). A new approach for the analysis of mass spectrometry data for biomarker discovery. American Medical Informatics Association Symmposium: November 11–15 2006 Washington DC, USA.

[B11] Frank R, Hargreaves R (2003). Clinical biomarkers in drug discovery and development. Nat Rev Drug Discov.

[B12] Cohen WW (1995). Fast effective rule induction. Proceedings of the Twelfth International Conference on Machine Learning: 1995.

[B13] Clearwater SH, Provost FJ (1990). RL4: A tool for knowledge-based induction. Proceedings of the Second International IEEE Conference on Tools for Artificial Intelligence (TAI-90): Nov 6–9, 1990 1990; Herndon, VA.

[B14] Buchanan BG, Livingston GR (2004). Toward automated discovery in the biological sciences. AI Magazine.

[B15] Gopalakrishnan V, Livingston GR, Hennessy D, Buchanan B, Rosenberg J (2004). Machine-learning techniques for macromolecular crystallization data. Acta Crystallographica Section D.

[B16] Dietterich T, Kong EB (1995). Machine learning bias, statistical bias, and statistical variance of decision tree algorithms. Oregon State University, Corvallis, OR, Tech Rep 97331-3202.

[B17] Fayyad UM, Irani KB (1993). Multi-interval discretization of continuous-valued attributes for classification learning. Proceedings of the Thirteenth International Joint Conference on AI (IJCAI-93): 1993; Chamberry, France.

[B18] Kohavi R, Sahami M (1996). Error-Based and Entropy-Based discretization of continuous features. Proceedings of the Second International Conference on Knowledge Discovery and Data Mining: 1996.

[B19] Sindhwani V, Bhattacharya P, Rakshit S (2001). Information theoretic feature crediting in multiclass support vector machines. Proceedings of the First SIAM International Conference on Data Mining: April 5–7th 2001; Chicago, IL.

